# EASIX for Long-Term Prognostication in Hospitalized Heart Failure with Preserved Ejection Fraction: Incremental Value Beyond Clinical Risk Markers

**DOI:** 10.3390/biomedicines14102228

**Published:** 2026-10-01

**Authors:** Çetin Alak, Kaan Turhan, Fazıl Çağrı Hunutlu, Zeynep Kumral, Tunay Şentürk

**Affiliations:** 1 Department of Cardiology, Faculty of Medicine, Bursa Uludag University, 16059 Bursa, Türkiye; kaanturhan@uludag.edu.tr (K.T.); tunaysenturk@uludag.edu.tr (T.Ş.); 2 Division of Hematology, Department of Internal Medicine, Bursa Sehir Training and Research Hospital, University of Health Sciences, 16009 Bursa, Türkiye; fazilhunutlu@gmail.com; 3 Department of Cardiology, Unye State Hospital, 52300 Ordu, Türkiye; zeynepkumral@gmail.com

**Keywords:** heart failure with preserved ejection fraction, endothelial activation and stress index, prognostic nutritional index, CONUT score, all-cause mortality, prognostic biomarkers, risk stratification

## Abstract

**Background/Objectives**: Heart failure with preserved ejection fraction (HFpEF) is a heterogeneous multisystem syndrome in which conventional cardiac measures may not fully capture systemic vulnerability. The Endothelial Activation and Stress Index (EASIX) is prognostic in other cardiovascular populations, but evidence in HFpEF remains limited. We evaluated the association of EASIX with long-term mortality and compared its prognostic value with the Prognostic Nutritional Index (PNI) and Controlling Nutritional Status (CONUT) score. **Methods**: This retrospective single-center study included 79 adults hospitalized with HFpEF between 2019 and 2025. EASIX, PNI, and CONUT were calculated from admission laboratory values. The endpoint was all-cause mortality. EASIX was modeled using restricted cubic splines. Incremental value beyond age, serum sodium, and B-type natriuretic peptide was assessed using likelihood-ratio tests. Harrell’s C-index was internally validated using 1000 bootstrap resamples; conventional and time-dependent receiver operating characteristic analyses were also performed. **Results**: During a median follow-up of 27.6 months, 45 patients (57.0%) died. EASIX was nonlinearly associated with mortality (overall and nonlinearity *p* < 0.001) and improved clinical model fit (Δχ^2^ = 24.36; *p* < 0.001). PNI improved the clinical model without EASIX (Δχ^2^ = 8.74; *p* = 0.003), but its contribution after EASIX was attenuated (Δχ^2^ = 3.54; *p* = 0.060); CONUT did not improve model fit (*p* = 0.859). Optimism-corrected C-indices were 0.728 for clinical + EASIX and 0.732 after adding PNI. Conventional AUCs were 0.753, 0.782, and 0.661 for EASIX, PNI, and CONUT, respectively. **Conclusions**: In this selected hospitalized HFpEF cohort, EASIX provided incremental prognostic information beyond clinical markers. These exploratory findings require confirmation in larger prospective cohorts with external validation.

## 1. Introduction

Heart failure with preserved ejection fraction (HFpEF) accounts for a substantial and increasing proportion of the overall heart failure burden and remains associated with frequent hospitalization and considerable long-term mortality [1]. Rather than representing a single cardiac phenotype, HFpEF is increasingly recognized as a heterogeneous, multisystem syndrome in which cardiovascular and non-cardiovascular comorbidities interact with metabolic, renal, inflammatory, and vascular abnormalities [1,2]. This systemic complexity is particularly relevant to prognosis. In a recent patient-level analysis of TOPCAT and PARAGON-HF, more than 95% of patients with mildly reduced or preserved ejection fraction had at least two major comorbidities, and several comorbidity combinations conferred risk beyond that expected from their individual effects [2]. Consequently, risk assessment based predominantly on conventional measures of cardiac dysfunction may incompletely characterize the broader biological vulnerability of patients with HFpEF.

Systemic inflammation and microvascular endothelial perturbation constitute one important mechanistic axis within this broader phenotype. The paradigm proposed by Paulus and Tschöpe linked common HFpEF comorbidities to a systemic proinflammatory state, coronary microvascular endothelial inflammation, impaired nitric oxide signaling, and subsequent myocardial stiffening and remodeling [3]. Subsequent clinical observations have provided support for the relevance of this pathway in human HFpEF. In the prospective, multicenter PROMIS-HFpEF study, coronary microvascular dysfunction was identified in approximately three quarters of patients despite the absence of unrevascularized epicardial coronary artery disease and was associated with systemic endothelial dysfunction, higher NT-proBNP concentrations, and impaired right ventricular function [4]. These findings reinforce the concept that vascular and systemic abnormalities may convey clinically relevant information that is not fully captured by left ventricular ejection fraction alone.

Nutritional and immunologic vulnerability represents another prognostically relevant dimension of HFpEF. Easily derived indices based on routine laboratory measurements, particularly the Prognostic Nutritional Index (PNI) and the Controlling Nutritional Status (CONUT) score, have been associated with adverse outcomes across both ambulatory and hospitalized HFpEF populations [5,6,7,8]. PNI has been associated with mortality and heart failure hospitalization and has provided prognostic information beyond natriuretic peptides in ambulatory HFpEF [6], whereas CONUT has been associated with subsequent cardiovascular events in prospective HFpEF cohorts [7]. However, comparative studies have yielded variable results regarding the relative prognostic performance of individual nutritional indices [5,8], suggesting that these measures capture overlapping but not necessarily interchangeable aspects of nutritional and immunologic reserve. This heterogeneity also raises the question of whether prognostic information derived from nutritional indices overlaps with, or is complementary to, markers reflecting other systemic disease processes.

The Endothelial Activation and Stress Index (EASIX), calculated from lactate dehydrogenase, serum creatinine, and platelet count, was originally developed as a readily available prognostic index in patients with acute graft-versus-host disease after allogeneic stem-cell transplantation [9]. Its prognostic relevance has subsequently extended beyond hematologic disease to cardiovascular populations, including patients with coronary artery disease and chronic heart failure [10,11]. Importantly, the large chronic heart failure validation study of EASIX was restricted to patients with reduced or mildly reduced ejection fraction and specifically excluded those with LVEF > 50% [11]; more recently, EASIX has also been associated with in-hospital mortality in patients hospitalized with acute decompensated heart failure with reduced ejection fraction [12]. Thus, despite a strong biological rationale for studying systemic and microvascular stress in HFpEF, the long-term prognostic significance of EASIX in a dedicated HFpEF population remains undefined. Moreover, whether the information conveyed by EASIX overlaps with or extends beyond established immunonutritional indices has not been directly examined. PNI and CONUT were therefore selected not as mechanistically equivalent comparators to EASIX, but as clinically accessible indices of systemic vulnerability with established prognostic relevance in HFpEF. Their inclusion allowed us to examine whether EASIX provides prognostic information that is distinct from, or overlaps with, that captured by immunonutritional indices.

Accordingly, we evaluated the association of EASIX with long-term all-cause mortality in patients hospitalized with HFpEF and compared its prognostic performance with PNI and CONUT. We further examined the functional form of the EASIX–mortality relationship and assessed the incremental prognostic information provided by EASIX and the nutritional indices beyond a parsimonious clinical risk model, with internal validation of the principal multivariable models.

## 2. Materials and Methods

### 2.1. Study Design and Population

This retrospective, observational, single-center cohort study included adult patients hospitalized at the Department of Cardiology, Bursa Uludağ University Faculty of Medicine, between 1 January 2019, and 1 January 2025.

A total of 1879 heart failure-related hospitalizations were identified during the study period. For patients with multiple HF-related hospitalizations at our center during the study period, the first qualifying hospitalization was defined as the index hospitalization and retained for analysis; subsequent admissions were not treated as additional observations. After restricting the dataset to one index hospitalization per patient and excluding patients with a left ventricular ejection fraction (LVEF) < 50%, 540 patients underwent detailed eligibility assessment**.**

Patients were eligible if they were aged ≥18 years and had a clinical phenotype consistent with hospitalized heart failure with preserved ejection fraction (HFpEF), defined by an LVEF ≥ 50%, symptoms and/or signs consistent with heart failure, a BNP concentration > 100 pg/mL, and clinical evidence of congestion requiring intravenous diuretic therapy during the index hospitalization [13]. BNP, rather than NT-proBNP, was used throughout the study period. The diagnosis of HFpEF was established by the treating cardiology team after integration of the clinical presentation, laboratory findings, and available echocardiographic findings, consistent with contemporary ESC diagnostic principles for HFpEF [14]. Detailed structural and diastolic echocardiographic variables, including E/e′, left atrial volume index, LV mass index, tricuspid regurgitation velocity/pulmonary artery systolic pressure, and formal diastolic dysfunction grade, were not consistently available across the study period. Body mass index was also not uniformly available. Consequently, H2FPEF and HFA-PEFF scores could not be systematically and reliably reconstructed.

To reduce phenotypic heterogeneity and minimize major competing causes of congestion or biomarker abnormalities, patients with alternative cardiac or systemic conditions that could substantially account for the index presentation were excluded. These included pulmonary vascular or major right-sided cardiac disease; severe valvular or structural heart disease, including severe tricuspid regurgitation; acute coronary syndrome or clinically relevant myocardial injury; and clinically significant arrhythmic or conduction-related conditions that accounted for the index hospitalization (e.g., atrial fibrillation with rapid or slow ventricular response, supraventricular tachycardia, ventricular tachycardia, or complete atrioventricular block). Active infectious or inflammatory disease, clinically significant pericardial disease, malignancy or hematologic malignancy, end-stage kidney disease requiring dialysis, and other major competing systemic conditions were also excluded. Pulmonary vascular exclusions comprised pulmonary arterial hypertension, clinically relevant precapillary pulmonary hypertension, and pulmonary embolic disease when these represented a major alternative mechanism of right-sided pressure or volume overload. Patients with infiltrative or inflammatory cardiomyopathy, advanced pulmonary or hepatic disease considered sufficient to explain the presenting syndrome, and other conditions judged to substantially confound the HFpEF phenotype were also excluded.

Following detailed clinical and etiologic review, 119 patients remained clinically eligible. Of these, 40 were excluded because one or more laboratory variables required for calculation of EASIX, PNI, or CONUT were unavailable. The final analytic cohort therefore comprised 79 patients with complete data for the variables required to calculate all three indices. The detailed patient-selection process and distribution of exclusion criteria are shown in Figure 1.

### 2.2. Data Collection

Demographic characteristics, cardiovascular risk factors, comorbidities, clinical characteristics, echocardiographic findings, laboratory measurements, hospitalization data, and follow-up information were retrospectively extracted from the institutional electronic medical record system.

Recorded clinical variables included age, sex, hypertension, diabetes mellitus, dyslipidemia, coronary artery disease, smoking status, atrial fibrillation, chronic obstructive pulmonary disease or asthma, previous stroke, chronic kidney disease, New York Heart Association (NYHA) functional class, and LVEF. Smoking was defined as current smoking at the time of the index hospitalization.

Available pharmacological treatment data from the index hospitalization included beta-blockers, renin–angiotensin system inhibitors or angiotensin receptor–neprilysin inhibitors (RAASi/ARNI), mineralocorticoid receptor antagonists (MRAs), and sodium–glucose cotransporter 2 (SGLT2) inhibitors.

Laboratory variables included BNP, serum sodium, chloride, creatinine, estimated glomerular filtration rate (eGFR), hemoglobin, lymphocyte count, platelet count, C-reactive protein (CRP), serum albumin, total cholesterol, and lactate dehydrogenase (LDH). EASIX, PNI, and CONUT were calculated using laboratory measurements obtained at admission. These indices were calculated only when all variables required for the respective index were available; missing laboratory values were not imputed.

### 2.3. Calculation of EASIX

EASIX was calculated from LDH, serum creatinine, and platelet count according to the following formula [9]:

EASIX = [LDH (U/L) × serum creatinine (mg/dL)]/platelet count (10^9^/L)

EASIX was analyzed primarily as a continuous variable. Its functional form in relation to mortality was examined before final model specification. Because the association was not adequately represented by a simple linear term, EASIX was modeled using a restricted cubic spline with three knots in the primary multivariable survival analyses. The spline was evaluated as an overall term, and its nonlinear component was assessed separately; individual spline-basis coefficients were not interpreted as clinically meaningful hazard ratios.

### 2.4. Calculation of the Prognostic Nutritional Index

PNI was calculated from serum albumin and peripheral blood lymphocyte count according to the following formula [15]:

PNI = albumin (g/L) + 5 × lymphocyte count (10^9^/L)

Higher PNI values indicate better nutritional and immunologic reserve. PNI was analyzed as a continuous variable and was scaled per 5-point increase in Cox regression analyses to facilitate clinical interpretation of the corresponding hazard ratio.

### 2.5. Calculation of the CONUT Score

The CONUT score was calculated using serum albumin, total cholesterol, and peripheral blood lymphocyte count according to the conventional scoring system [16].

For serum albumin, 0, 2, 4, and 6 points were assigned for concentrations ≥ 35, 30–34.9, 25–29.9, and <25 g/L, respectively. For lymphocyte count, 0, 1, 2, and 3 points were assigned for values ≥ 1.6, 1.2–1.59, 0.8–1.19, and <0.8 × 10^9^/L, respectively. For total cholesterol, 0, 1, 2, and 3 points were assigned for concentrations ≥ 180, 140–179, 100–139, and <100 mg/dL, respectively. Component scores were summed to obtain the total CONUT score, ranging from 0 to 12.

For descriptive purposes, conventional nutritional categories were defined as normal nutritional status (0–1 points), mild nutritional impairment (2–4 points), moderate nutritional impairment (5–8 points), and severe nutritional impairment (9–12 points). Because few patients were represented in the higher categories, CONUT was retained as a continuous variable for the principal survival analyses.

### 2.6. Study Endpoint and Follow-Up

The primary endpoint was long-term all-cause mortality.

Follow-up began at the index HFpEF hospitalization. For patients who died, follow-up extended from the index hospitalization to the date of death; patients who remained alive were censored at the date of their last documented clinical contact. Time-to-event analyses were performed using follow-up duration in days, whereas time was expressed in years for graphical presentation of Kaplan–Meier survival curves. Vital status was available for all patients included in the final analytic cohort.

### 2.7. Statistical Analysis

All final statistical analyses were performed using R statistical software, version 4.6.1 (R Foundation for Statistical Computing, Vienna, Austria). The distribution of continuous variables was assessed using the Shapiro–Wilk test together with visual inspection of Q–Q plots. Approximately normally distributed variables were presented as mean ± standard deviation, whereas non-normally distributed variables were presented as median with first and third quartiles [Q1, Q3]. Categorical variables were presented as numbers and percentages. Baseline characteristics were compared between survivors and non-survivors using Welch’s two-sample *t*-test for approximately normally distributed continuous variables and the Wilcoxon rank-sum test for non-normally distributed variables. Fisher’s exact test was used for categorical comparisons, as appropriate.

Associations among EASIX, PNI, and CONUT were evaluated using Spearman rank correlation coefficients. Ninety-five percent confidence intervals for the correlation coefficients were estimated using nonparametric bootstrap resampling with 5000 replicates. The correlation between PNI and CONUT was specifically examined before multivariable modeling because the two indices share nutritional and immunologic components.

#### 2.7.1. Cox Proportional Hazards Analysis

Associations between candidate clinical, laboratory, and nutritional variables and all-cause mortality were initially examined using univariable Cox proportional hazards regression. Hazard ratios (HRs) are reported with 95% confidence intervals (CIs).

To facilitate clinical interpretation, selected continuous variables were rescaled before regression analysis. BNP was expressed per 100-pg/mL increase, eGFR per 10-mL/min/1.73 m^2^ increase, platelet count per 50 × 10^9^/L increase, CRP per 10-mg/L increase, albumin per 5-g/L increase, total cholesterol per 10-mg/dL increase, LDH per 100-U/L increase, and PNI per 5-point increase. These increments were selected solely to facilitate interpretation of the hazard ratios and were not data-derived or intended to represent clinically validated thresholds; linear rescaling changes the reporting unit of the effect estimate but does not alter model fit or statistical inference.

The functional form of the principal continuous prognostic markers was examined before final multivariable modeling using restricted cubic splines with three knots. EASIX demonstrated evidence of nonlinearity and was therefore retained as a three-knot restricted cubic spline in the final models, whereas PNI was adequately represented as a linear continuous predictor.

Given the number of observed deaths relative to the cohort size, a deliberately parsimonious multivariable modeling strategy was adopted to limit overfitting. The clinical reference model was specified a priori on clinical grounds and was not derived from univariable significance testing or automated stepwise selection. It comprised age, serum sodium, and BNP, representing complementary domains of demographic risk, systemic/hemodynamic severity, and cardiac wall stress, respectively. These variables are routinely available in hospitalized patients with HFpEF and were selected to provide clinically interpretable baseline adjustment while avoiding direct inclusion of variables that constitute or substantially overlap with EASIX, PNI, or CONUT. The primary prognostic model then added spline-modeled EASIX to this prespecified clinical reference model.

An extended model additionally incorporated PNI to assess whether nutritional reserve provided incremental prognostic information beyond the clinical variables and EASIX. PNI was also added separately to the clinical reference model to quantify its contribution before inclusion of EASIX. As an alternative nutritional comparator, CONUT was added to the clinical + EASIX model. PNI and CONUT were not simultaneously entered into the principal multivariable models because of their substantial biological and statistical overlap.

Nested models were compared using likelihood-ratio tests. The overall contribution of EASIX and the presence of nonlinearity were evaluated through joint testing of the restricted-cubic-spline terms rather than interpretation of individual spline-basis coefficients.

#### 2.7.2. Proportional Hazards Assumption and Sensitivity Analyses

The proportional hazards assumption was evaluated using scaled Schoenfeld residuals for individual model terms and for the global model.

Potential departures from proportional hazards for spline-modeled EASIX were further examined in a sensitivity analysis allowing the EASIX spline components to interact with log-transformed follow-up time. The fit of this time-varying coefficient model was compared with that of the corresponding proportional-hazards model using a likelihood-ratio test.

The robustness of the nonlinear EASIX–mortality association was additionally examined after exclusion of the single highest EASIX observation and, separately, after exclusion of observations above the 95th percentile of the EASIX distribution.

To assess whether the prognostic information provided by EASIX was primarily driven by its individual components, additional sensitivity models compared EASIX with creatinine, LDH, and platelet count, as well as eGFR as an alternative marker of renal function, using the same clinical reference model. Functional forms were evaluated before model specification; creatinine was modeled using a three-knot restricted cubic spline, whereas eGFR, LDH, and platelet count were retained as linear terms. Model contribution was compared using likelihood-ratio tests, AIC, and Harrell’s C-index. Bidirectional analyses evaluated the incremental contribution of EASIX after inclusion of each component or eGFR in the clinical reference model and, conversely, the contribution of each variable after inclusion of EASIX. Finally, a model containing creatinine, LDH, and platelet count as separate predictors was compared with the clinical + EASIX model.

To examine potential temporal effects related to changes in HFpEF management during the study period, an additional sensitivity model included calendar year of the index hospitalization as a covariate in the primary clinical + EASIX model.

Given the unexpected inverse association between current smoking and mortality in univariable analysis, an additional sensitivity analysis included current smoking as a covariate in the primary clinical + EASIX model.

#### 2.7.3. Model Performance and Internal Validation

Model discrimination was quantified using Harrell’s concordance index (C-index), and relative model fit was additionally summarized using the Akaike information criterion (AIC).

Internal validation was performed using 1000 bootstrap resamples to estimate optimism resulting from model development and evaluation within the same cohort. Apparent and optimism-corrected C-indices and optimism-corrected calibration slopes were obtained for the primary clinical + EASIX model and the extended clinical + EASIX + PNI model. Calibration was additionally assessed graphically at 3 years by comparing model-predicted survival probabilities with apparent Kaplan–Meier estimates and bootstrap optimism-corrected calibration estimates of observed survival across approximately equal-sized groups of predicted survival. Three years was selected as the graphical calibration horizon because it represented a clinically relevant long-term time point while retaining substantially more patients at risk than at 5 years.

Model selection emphasized parsimony, incremental likelihood-ratio testing, and internally validated performance rather than significance-based stepwise variable selection.

#### 2.7.4. Conventional ROC Analysis

Conventional receiver operating characteristic (ROC) analysis was performed for EASIX, PNI, and CONUT as a complementary assessment of discrimination for overall mortality status. Areas under the curve (AUCs) and corresponding 95% CIs were estimated using the DeLong method, and pairwise differences between correlated AUCs were assessed using paired DeLong tests.

Optimal thresholds were identified using the Youden index. Because CONUT is integer-valued, a mathematical ROC threshold falling between two adjacent scores was expressed using the corresponding clinically applicable integer cutoff.

Because conventional ROC analysis does not account for censoring or variation in follow-up duration, these analyses were considered complementary to the survival-based assessments of prognostic discrimination.

#### 2.7.5. Time-Dependent ROC Analysis

Time-dependent ROC analyses were performed at 1, 3, and 5 years to evaluate discrimination while accounting for censoring and differences in follow-up duration. Time-dependent AUCs were estimated using inverse probability of censoring weighting with the marginal estimator.

Because lower PNI values indicate greater risk, the direction of PNI was reversed for time-dependent ROC calculations so that higher transformed marker values consistently represented greater predicted risk.

Pairwise time-dependent AUC comparisons were performed for EASIX versus PNI, EASIX versus CONUT, and PNI versus CONUT. To account for repeated testing across the three time points, *p* values were adjusted using the Holm method within each marker pair. As a more conservative sensitivity analysis, the Holm procedure was also applied jointly across all nine marker-by-time comparisons.

The number of patients remaining at risk at each time point was considered when interpreting late time-dependent discrimination estimates.

#### 2.7.6. Kaplan–Meier Analysis

Kaplan–Meier survival analyses were performed as exploratory graphical assessments using thresholds derived from the conventional ROC analyses. Patients were categorized as having low EASIX (<1.08) or high EASIX (≥1.08) and as having high PNI (≥47.6) or low PNI (<47.6). Survival distributions were compared using the log-rank test, and numbers at risk were displayed below each Kaplan–Meier curve.

One-, three-, and five-year Kaplan–Meier survival estimates were calculated for each group. Because these thresholds were derived and evaluated within the same study population, the dichotomized analyses were considered exploratory and were not interpreted as externally validated clinical decision thresholds.

All statistical tests were two-sided. A *p* value < 0.05 was considered statistically significant unless adjustment for multiple comparisons was applied, in which case the corresponding adjusted *p* value was used.

### 2.8. Ethical Considerations

The study was conducted in accordance with the Declaration of Helsinki and was approved by the Bursa Uludağ University Health Research Ethics Committee on 10 June 2026 (decision no. 2026/311/11-15).

## 3. Results

### 3.1. Study Population and Baseline Characteristics

The final analytic cohort comprised 79 patients hospitalized with HFpEF. During a median follow-up of 27.6 months (IQR, 10.5–44.4 months), 45 patients (57.0%) died. The mean age was 72.9 ± 10.4 years, 49% were women, and mean LVEF was 56.8 ± 4.2%. Pharmacological treatment patterns during the index hospitalization are summarized in Table 1. Beta-blockers were used in 68.4% of patients, RAASi/ARNI in 54.4%, MRAs in 16.5%, and SGLT2 inhibitors in 11.4%.

The three indices of interest differed significantly according to vital status. EASIX was substantially higher in non-survivors than in survivors (1.39 [0.89–2.01] vs. 0.72 [0.56–0.99]; *p* < 0.001), whereas PNI was lower (42.9 ± 6.5 vs. 50.4 ± 7.4; *p* < 0.001). CONUT scores were also higher among non-survivors (3 [1–4] vs. 1 [1–3]; p = 0.013). Non-survivors additionally had higher BNP, creatinine, CRP, and LDH concentrations and lower eGFR and albumin (Table 1).

PNI and CONUT were inversely correlated (Spearman ρ = −0.806, 95% CI −0.881 to −0.698; *p* < 0.001). By contrast, EASIX was only weakly correlated with PNI (ρ = −0.202, 95% CI −0.395 to 0.014; *p* = 0.074) and showed no meaningful correlation with CONUT (ρ = 0.089, 95% CI −0.123 to 0.319; *p* = 0.435).

### 3.2. Univariable Prognostic Associations

Complete univariable Cox regression results are presented in Table 2. All three indices were associated with all-cause mortality when initially evaluated as conventional continuous predictors. EASIX was associated with a higher hazard of death (HR 1.31 per 1-unit increase, 95% CI 1.08–1.60; *p* = 0.007), whereas higher PNI was associated with a lower hazard (HR 0.63 per 5-point increase, 95% CI 0.51–0.78; *p* < 0.001). CONUT was likewise associated with mortality (HR 1.21 per 1-point increase, 95% CI 1.05–1.39; *p* = 0.009).

The conventional linear estimate for EASIX was not carried forward as the principal measure of association because formal assessment of functional form demonstrated a marked departure from linearity.

The apparently inverse association between current smoking and mortality observed in univariable analysis was attenuated after adjustment for the primary clinical model and EASIX and was no longer statistically significant (HR 0.60, 95% CI 0.17–2.07; *p* = 0.419).

### 3.3. Nonlinear Association Between EASIX and Mortality

Restricted cubic spline analysis demonstrated a nonlinear association between EASIX and all-cause mortality (overall χ^2^ = 20.18, 2 df; *p* < 0.001; *p* for nonlinearity < 0.001) (Figure 2). Relative to the median EASIX value, the estimated hazard increased steeply across the lower-to-intermediate range of EASIX. Confidence intervals widened toward the upper end of the distribution, where observations were sparse; the apparent downturn in the point estimate at very high EASIX values therefore occurred in a region of substantially greater uncertainty.

The nonlinear association persisted in sensitivity analyses. After exclusion of the single highest EASIX value, both the overall association and nonlinear component remained significant (both *p* < 0.001). Similar findings were obtained after exclusion of the upper 5% of the EASIX distribution (overall *p* < 0.001; *p* for nonlinearity = 0.003). By contrast, PNI showed no evidence of departure from linearity (*p* for nonlinearity = 0.257) and was therefore retained as a linear continuous predictor.

### 3.4. Incremental Prognostic Value and Multivariable Modeling

The clinical reference model comprised age, sodium, and BNP. Addition of spline-modeled EASIX improved model fit (Δχ^2^ = 24.36, 2 df; *p* < 0.001). In the resulting primary model, both the overall EASIX term and its nonlinear component remained associated with mortality after multivariable adjustment (both *p* < 0.001) (Table 3). Estimates for the clinical adjustment variables are reported in Table 3.

PNI also improved the clinical reference model when evaluated in the absence of EASIX (Δχ^2^ = 8.74, 1 df; *p* = 0.003). This contribution was substantially attenuated once EASIX was incorporated: addition of PNI to the clinical + EASIX model yielded Δχ^2^ = 3.54 (1 df; *p* = 0.060). Consistent with this result, the inverse association with PNI in the extended model did not reach conventional statistical significance (HR 0.78 per 5-point increase, 95% CI 0.59–1.02; *p* = 0.066). Conversely, addition of EASIX to a model already containing PNI resulted in a significant improvement in model fit (Δχ^2^ = 19.16, 2 df; *p* < 0.001).

CONUT showed a different pattern. Despite its univariable association with mortality, addition of CONUT to the clinical + EASIX model produced essentially no improvement in model fit (Δχ^2^ = 0.03, 1 df; *p* = 0.859).

In component-level sensitivity analyses, EASIX provided a larger improvement over the clinical reference model than any individual component or eGFR as an alternative marker of renal function. Addition of EASIX improved model fit by Δχ^2^ = 24.36 (2 df; *p* < 0.001), with an AIC of 317.25 and C-index of 0.763. Corresponding values were Δχ^2^ = 14.26 (2 df; *p* < 0.001), AIC = 327.35, and C-index = 0.708 for creatinine; Δχ^2^ = 9.06 (1 df; *p* = 0.003), AIC = 330.56, and C-index = 0.688 for eGFR; Δχ^2^ = 9.38 (1 df; *p* = 0.002), AIC = 330.24, and C-index = 0.743 for LDH; and Δχ^2^ = 2.00 (1 df; *p* = 0.158), AIC = 337.62, and C-index = 0.673 for platelet count.

When creatinine, LDH, and platelet count were entered as separate predictors in addition to the clinical reference model, the likelihood-ratio improvement was similar to that obtained with EASIX (Δχ^2^ = 24.82 vs. 24.36), but the component model required four biomarker degrees of freedom and had a higher AIC (320.79 vs. 317.25). Its apparent C-index was only marginally higher than that of the clinical + EASIX model (0.771 vs. 0.763). In bidirectional sensitivity analyses, EASIX remained informative when added to models already containing creatinine (Δχ^2^ = 11.08, 2 df; *p* = 0.004), eGFR (Δχ^2^ = 15.69, 2 df; *p* < 0.001), LDH (Δχ^2^ = 16.24, 2 df; *p* < 0.001), or platelet count (Δχ^2^ = 27.29, 2 df; *p* < 0.001). Conversely, addition of creatinine (Δχ^2^ = 0.98, 2 df; *p* = 0.612), eGFR (Δχ^2^ = 0.38, 1 df; *p* = 0.537), or LDH (Δχ^2^ = 1.25, 1 df; *p* = 0.263) after EASIX did not materially improve model fit, whereas platelet count retained a smaller residual contribution (Δχ^2^ = 4.93, 1 df; *p* = 0.026).

Scaled Schoenfeld residual testing suggested possible non-proportionality for the spline-modeled EASIX term (*p* = 0.044), while the global test was borderline (*p* = 0.052). A sensitivity model allowing the EASIX spline components to vary with follow-up time did not significantly improve model fit (likelihood-ratio *p* = 0.359).

Additional adjustment for calendar year of the index hospitalization did not materially alter the primary model. Calendar year was not statistically associated with mortality (HR 1.04, 95% CI 0.85–1.28; *p* = 0.680), and its inclusion did not improve model fit (likelihood-ratio *p* = 0.679) or materially change discrimination (C-index from 0.763 to 0.767).

### 3.5. Internal Validation and Model Performance

The apparent Harrell C-index of the primary clinical + EASIX model was 0.763. After 1000 bootstrap resamples, the optimism-corrected C-index was 0.728, with an optimism-corrected slope of 0.729. The extended clinical + EASIX + PNI model yielded an apparent C-index of 0.774 and an optimism-corrected C-index of 0.732, with a corrected slope of 0.667.

Thus, although inclusion of PNI increased apparent discrimination by approximately 0.011, the corresponding gain after correction for optimism was only approximately 0.004. Together with the likelihood-ratio results and the greater shrinkage observed in the extended model, these findings supported retention of the clinical + EASIX model as the more parsimonious specification.

Three-year calibration plots for the primary and extended models are presented in Supplementary Figure S1. Calibration was imperfect, particularly in the intermediate predicted-survival range, and this pattern remained evident after bootstrap correction for optimism.

### 3.6. Kaplan–Meier Survival Analysis

Kaplan–Meier curves showed marked separation between the high- and low-EASIX groups (log-rank *p* < 0.001) (Figure 3). Estimated survival at 1, 3, and 5 years was 56.8%, 27.9%, and 12.5%, respectively, among patients with EASIX ≥ 1.08, compared with 88.1%, 75.9%, and 68.6% among those with EASIX < 1.08.

Survival also differed substantially according to PNI (log-rank *p* < 0.001). Estimated survival at 1, 3, and 5 years was 66.0%, 37.4%, and 23.2%, respectively, among patients with PNI < 47.6, compared with 84.4%, 77.2%, and 71.7% among those with PNI ≥ 47.6.

Because both thresholds were derived within the present cohort, these dichotomized analyses were considered exploratory and were used to illustrate risk separation rather than to establish externally validated clinical cut points.

### 3.7. Conventional Discrimination

Conventional ROC analysis showed the numerically highest AUC for PNI (0.782, 95% CI 0.675–0.889), followed by EASIX (0.753, 95% CI 0.634–0.872) and CONUT (0.661, 95% CI 0.539–0.782) (Table 4; Figure 4). The Youden-derived thresholds were 1.078 for EASIX, 47.625 for PNI, and ≥2 for CONUT.

Despite its numerically higher AUC, PNI did not differ significantly from EASIX on paired DeLong testing (absolute ΔAUC = 0.029; *p* = 0.719). The EASIX–CONUT comparison was likewise non-significant (*p* = 0.273). By contrast, PNI showed significantly greater discrimination than CONUT (ΔAUC = 0.121, 95% CI 0.043–0.200; *p* = 0.002).

### 3.8. Time-Dependent Discrimination

Time-dependent ROC analysis provided a censoring-aware assessment of discrimination at 1, 3, and 5 years (Table 5). EASIX showed AUCs of 0.745 (95% CI 0.624–0.867), 0.760 (95% CI 0.635–0.884), and 0.803 (95% CI 0.620–0.986), respectively. Corresponding AUCs for PNI were 0.613 (95% CI 0.485–0.742), 0.725 (95% CI 0.604–0.847), and 0.805 (95% CI 0.676–0.934); those for CONUT were 0.516 (95% CI 0.365–0.667), 0.607 (95% CI 0.474–0.739), and 0.641 (95% CI 0.480–0.802).

EASIX and PNI did not differ significantly at any of the three time points after adjustment for multiple comparisons across time within the marker pair. PNI showed greater discrimination than CONUT at 3 years (adjusted *p* = 0.024) and 5 years (adjusted *p* = 0.002). However, when all nine time-specific pairwise comparisons were considered jointly using the more conservative Holm procedure, only the 5-year PNI–CONUT comparison remained significant (adjusted *p* = 0.008), whereas the 3-year comparison was attenuated to *p* = 0.075. Because only 12 patients remained at risk at 5 years, the 5-year time-dependent AUC estimates were considered exploratory and should be interpreted with substantial caution because of the limited information available at this late time point.

## 4. Discussion

In this study of patients hospitalized with HFpEF, EASIX was associated with long-term all-cause mortality and provided prognostic information beyond a parsimonious clinical model incorporating age, serum sodium, and BNP. Several findings merit particular emphasis. First, the relationship between EASIX and mortality was nonlinear and persisted after exclusion of extreme observations, indicating that its prognostic information was not adequately represented by a single linear effect estimate. Second, EASIX was only weakly correlated with PNI and showed no meaningful correlation with CONUT, whereas PNI and CONUT were inversely correlated with each other, suggesting that EASIX may capture a risk dimension that is at least partly distinct from conventional immunonutritional assessment. Third, although PNI provided incremental information when added to the clinical reference model, its contribution was attenuated after EASIX was incorporated, whereas CONUT provided no additional model improvement. Conversely, EASIX continued to improve model fit when added to a model already containing PNI. Finally, PNI and EASIX showed comparable overall and time-dependent discrimination, underscoring that the present findings should not be interpreted as demonstrating superiority of one marker over the other. Rather, they suggest that EASIX and immunonutritional indices may characterize different, partly overlapping dimensions of vulnerability, with EASIX providing incremental prognostic information in multivariable models beyond conventional clinical markers.

EASIX was initially developed outside cardiovascular medicine as a composite of LDH, creatinine, and platelet count associated with adverse outcomes following allogeneic stem-cell transplantation [9]. Subsequent studies broadened its prognostic relevance to cardiovascular disease. Finke et al. demonstrated an association between EASIX and long-term mortality in patients undergoing coronary evaluation, with consistent findings in an independent validation cohort [10]. More directly relevant to heart failure, Estler et al. evaluated 3592 patients with chronic HF in separate training and validation cohorts and found that EASIX remained associated with 5-year mortality after accounting for NT-proBNP, NYHA functional class, HF etiology, and other clinical characteristics [11]. Notably, that registry was restricted to HFrEF and HFmrEF, and patients with preserved or improved LVEF > 50% were explicitly excluded from the analysis [11]. More recently, Özlek et al. reported that admission EASIX predicted in-hospital mortality in 850 patients with acute decompensated HFrEF and added prognostic information to conventional clinical and laboratory models [12]. The present study therefore extends the emerging HF literature on EASIX into a clinically distinct setting: patients hospitalized with preserved LVEF and followed for long-term mortality rather than early mortality during an acute HFrEF admission. To our knowledge, EASIX has not previously been specifically evaluated for long-term prognosis in a dedicated HFpEF cohort.

An important feature of our findings was the nonlinear shape of the EASIX–mortality association. When EASIX was initially treated as a conventional linear predictor, higher values were associated with mortality; however, restricted cubic spline analysis demonstrated that this single HR incompletely described the relationship. Mortality risk rose steeply across the lower-to-intermediate range of EASIX, whereas estimates became increasingly uncertain at the upper extreme of the distribution (Figure 2). Importantly, evidence of nonlinearity persisted after removal of the highest EASIX observation and after exclusion of the upper 5% of values, arguing against a finding driven solely by isolated outliers. The apparent decline in the fitted hazard at very high EASIX values should nevertheless not be interpreted as a biological reversal of risk, because these estimates were derived from a sparsely populated region with wide confidence intervals. Previous HF work has also recognized that the relationship between EASIX and outcome may not be adequately characterized by simple linear assumptions; Estler et al. used flexible fractional-polynomial modeling, while nonlinear associations have also been explored in acute decompensated HFrEF [11,12]. Our findings reinforce the importance of examining functional form before reducing EASIX to either a per-unit HR or an arbitrary dichotomous threshold. The borderline proportional-hazards diagnostic observed in the present cohort warrants similar caution, although allowing the EASIX effect to vary over time did not improve model fit, supporting retention of the more parsimonious proportional-hazards specification.

The comparison with nutritional indices provides an additional perspective on the prognostic information carried by EASIX. Nutritional vulnerability is well established in HFpEF, but previous studies have not identified a uniformly dominant nutritional index. Chien et al. found that several nutritional measures, including PNI and CONUT, were associated with mortality in hospitalized HFpEF [5], while Zencirkiran Agus and Kahraman demonstrated that lower PNI predicted death or HF hospitalization in ambulatory HFpEF and provided information beyond NT-proBNP [6]. Komorita et al. subsequently showed an independent relationship between CONUT and cardiovascular events in a prospective cohort of 506 patients with HFpEF [7]. Conversely, direct comparisons have yielded different results across populations. Chen et al. reported higher prognostic performance for CONUT than for other objective nutritional indices in older patients with HFpEF [8]. In contrast, Fan et al. evaluated 218 elderly patients with HFpEF followed for approximately 5 years and found PNI to outperform CONUT for all-cause mortality (AUC 0.698 vs. 0.621), with low PNI independently associated with mortality [17]. Our conventional ROC results were directionally consistent with the latter study: PNI had greater discrimination than CONUT, although PNI and EASIX did not differ significantly.

The variability across studies is clinically informative rather than contradictory. In a recent cohort of 200 considerably older hospitalized patients with HFpEF (mean age 86.6 years), Sonaglioni et al. found that CONUT, but not PNI, remained independently associated with long-term mortality after multivariable adjustment, with higher discrimination for CONUT [18]. Together with the contrasting findings of Fan et al. and the present study, these data suggest that the relative performance of PNI and CONUT is likely phenotype-dependent and may vary with age, comorbidity burden, clinical setting, timing of laboratory assessment, and outcome definition. An additional methodological distinction may also contribute: PNI retains albumin and lymphocyte count as continuous variables, whereas CONUT converts albumin, lymphocyte count, and cholesterol into ordinal point categories, potentially sacrificing information within each category. This possibility is consistent with the direct PNI–CONUT comparison reported by Fan et al., but it cannot fully explain the heterogeneous findings across HFpEF cohorts and should not be interpreted as establishing intrinsic superiority of PNI.

Our multivariable analyses help clarify an issue that ROC comparisons alone cannot address. PNI was prognostically informative in its own right: it was associated with mortality in univariable analysis, separated Kaplan–Meier survival curves, and improved the clinical reference model when EASIX was absent. Its conventional AUC was, in fact, numerically the highest among the three indices. Nevertheless, discrimination of a marker considered in isolation and incremental information after accounting for other predictors are fundamentally different properties. Once EASIX had been incorporated into the clinical model, the additional contribution of PNI became modest, whereas EASIX continued to improve model fit when added to a model already containing PNI. CONUT provided essentially no incremental improvement after EASIX. The correlation structure offers a potential explanation: PNI and CONUT were inversely correlated (ρ = −0.806), consistent with their shared dependence on albumin and lymphocyte count, whereas EASIX showed only a weak relationship with PNI and no meaningful correlation with CONUT. These findings suggest that the indices are not interchangeable and may reflect partly distinct domains of clinical vulnerability.

The bootstrap results further support this interpretation while also emphasizing the need for restraint. Addition of PNI to the clinical + EASIX model increased the apparent C-index from 0.763 to 0.774, but the difference after optimism correction was only approximately 0.004, and the extended model showed greater shrinkage. In a modest-sized cohort, this pattern argues against retaining additional predictors solely on the basis of small apparent gains in discrimination within the development sample. Accordingly, the clinical + EASIX model was favored not because PNI lacked prognostic relevance, but because its incremental contribution beyond EASIX was limited after accounting for optimism. Taken together, these findings favor parsimony over biomarker competition.

The prognostic information provided by EASIX did not appear to be explained by any single component, including creatinine. EASIX provided a larger improvement in model fit than creatinine, LDH, platelet count, or eGFR when each was evaluated separately beyond the same clinical reference model. In bidirectional analyses, EASIX continued to improve model fit after inclusion of creatinine, eGFR, LDH, or platelet count, whereas creatinine, eGFR, and LDH did not provide meaningful additional improvement after EASIX; platelet count retained a smaller residual contribution. Modeling all three EASIX components separately yielded only a marginally higher apparent C-index than the clinical + EASIX model, despite requiring greater model complexity and resulting in a higher AIC. These findings support EASIX as a parsimonious composite representation of prognostic information rather than indicating that its association is predominantly attributable to renal dysfunction.

EASIX was calculated from admission laboratory values rather than measurements obtained after in-hospital decongestion or at discharge. Consequently, subsequent changes in creatinine during in-hospital diuretic therapy were not incorporated into the EASIX calculation. Nevertheless, because creatinine is an integral component of EASIX, admission values may reflect both chronic renal dysfunction and acute cardiorenal or hemodynamic stress, which should be considered when interpreting the prognostic information conveyed by the index.

The biological basis of the observed EASIX association warrants careful interpretation. EASIX should not be regarded as a direct measurement of endothelial function. Its components may instead integrate several consequences of systemic disease: LDH may reflect cellular injury and metabolic or hypoxic stress, creatinine reflects renal dysfunction and cardiorenal vulnerability, and platelet count may be influenced by inflammatory, thrombotic, hematologic, and endothelial processes. In HFpEF, this composite may be particularly relevant because disease progression is increasingly understood to involve interactions between systemic comorbidities, inflammation, microvascular dysfunction, and extracardiac organ impairment [1,3]. The Paulus–Tschöpe paradigm proposed a mechanistic link between comorbidity-driven systemic inflammation, coronary microvascular endothelial inflammation, and myocardial remodeling [3], while PROMIS-HFpEF subsequently demonstrated coronary microvascular dysfunction in approximately three quarters of a well-characterized HFpEF cohort and linked it to systemic endothelial dysfunction and markers of HF severity [4]. Our findings are biologically compatible with this framework, but they do not establish that EASIX specifically measures coronary microvascular or endothelial dysfunction. A more appropriate interpretation is that EASIX may act as an inexpensive integrative surrogate of systemic, cellular, renal, and vascular stress in a syndrome in which these processes frequently coexist.

Nutritional indices require similar caution in biological interpretation. Albumin, lymphocyte count, and cholesterol are influenced not only by nutritional intake but also by inflammation, congestion, hepatic function, catabolism, and disease severity. This is particularly relevant in hospitalized HFpEF. Large-scale data from PARAGON-HF showed that abnormal CONUT and/or GNRI scores, assessed longitudinally in 4794 patients, were associated with excess cardiovascular and noncardiovascular events; importantly, hospitalization itself increased the subsequent likelihood of developing an abnormal nutritional score, supporting a bidirectional relationship between HF-related illness and nutritional-risk markers [19]. Similar evidence comes from the prospective PURSUIT-HFpEF registry, in which deterioration of CONUT status from discharge to 1 year was associated with a substantially higher subsequent risk of death or HF readmission, whereas normalization was associated with lower risk; changes in CONUT also tracked with CRP, hemoglobin, and NT-proBNP [20]. These observations reinforce two implications of our findings. First, PNI and CONUT are better viewed as immunonutritional or nutritional-risk indices than as pure measures of caloric or protein deficiency. Second, a single measurement during an acute hospitalization provides a prognostic snapshot but cannot characterize the dynamic evolution of nutritional and inflammatory status after discharge.

The high mortality observed in the present cohort should be interpreted in the context of the study population. Importantly, the 57.0% proportion of deaths was observed over variable follow-up and should not be interpreted as a fixed 2-year mortality estimate. This was a selected hospitalized HFpEF cohort rather than an ambulatory or broadly representative HFpEF population; all patients had clinical congestion requiring intravenous diuretic therapy at the index hospitalization, and the cohort was relatively elderly, with a mean age of 72.9 years and a substantial burden of comorbidity. These characteristics identify a clinically vulnerable population in whom long-term mortality may therefore be higher than in more stable outpatient HFpEF cohorts or clinical-trial populations. Nevertheless, the observed event rate remains high and may also reflect the single-center, retrospective selection process. Accordingly, the absolute mortality rate and the magnitude of prognostic associations should not be directly extrapolated to broader or lower-risk HFpEF populations.

An apparently inverse association between current smoking and mortality was observed in univariable analysis. Given the small number of current smokers and the limited number of deaths in this subgroup, this counterintuitive finding is likely to be unstable and may reflect residual confounding or random variation rather than a protective effect of smoking. Consistent with this interpretation, the association was attenuated and no longer statistically significant after adjustment for the primary clinical model and EASIX. The use of all-cause mortality as the principal endpoint is also relevant to the multidomain nature of these markers. HFpEF is characterized by substantial noncardiovascular as well as cardiovascular mortality. In the long-term KaRen cohort, 68% of patients died during a median 5.4 years after an acute HFpEF presentation, with cardiovascular and noncardiovascular causes accounting for approximately equal proportions of deaths [21]. This broad competing-risk burden provides a rationale for evaluating markers that integrate systemic vulnerability rather than exclusively cardiac dysfunction. In the present cohort, EASIX improved prognostic modeling even after BNP was included, suggesting that the information it conveys is not simply a surrogate for conventional HF severity. Nevertheless, the current findings do not support use of EASIX as a replacement for natriuretic peptides or established clinical assessment. Its potential value is more plausibly complementary, particularly as all three components are routinely measured and require no additional testing or cost.

### Strengths and Limitations

Several features strengthen the present analysis. The study focused on a clinically selected hospitalized HFpEF phenotype after detailed exclusion of major alternative causes of congestion and biomarker abnormalities. Despite the modest sample size, the occurrence of 45 deaths permitted time-to-event analyses over long-term follow-up. Rather than relying solely on dichotomized biomarker cutoffs, the functional form of EASIX was formally examined, extreme-value sensitivity analyses were performed, and proportional-hazards assumptions were evaluated. Incremental prognostic information was assessed using nested models rather than stepwise significance-based selection, and apparent model performance was subjected to bootstrap correction for optimism. Finally, conventional ROC analysis was complemented by time-dependent discrimination that accounted for censoring and variable follow-up. Collectively, these approaches provide a more complete assessment of prognostic information than a single ROC curve or binary biomarker comparison. The study nevertheless has important limitations. First, its retrospective, single-center design and modest final sample size limit precision and external generalizability. Although model complexity was deliberately limited and bootstrap internal validation was performed, the number of observed events relative to the effective model degrees of freedom remained modest, corresponding to approximately 9 events per parameter in the primary model and 7.5 in the extended model. Accordingly, some degree of overfitting and coefficient instability cannot be excluded, particularly for the spline-modeled nonlinear EASIX effect. The optimism-corrected calibration slopes below 1.0, especially in the extended model, are consistent with shrinkage and further support cautious interpretation. Therefore, the exact magnitude and shape of the estimated associations require confirmation in larger cohorts with external validation.

The stringent clinical selection process produced a relatively homogeneous hospitalized HFpEF phenotype but may also limit applicability to patients with substantial valvular disease, advanced right-sided HF, pulmonary vascular disease, or other competing causes of congestion. Furthermore, 40 otherwise clinically eligible patients were excluded because one or more laboratory variables required for calculation of EASIX, PNI, or CONUT were unavailable. Because complete and consistently verifiable baseline data were not available for all of these excluded patients, a reliable comparison with the final analytic cohort could not be performed. This complete-case approach may therefore have introduced selection bias, and the included cohort may not fully represent the broader population of patients hospitalized with HFpEF. The findings should therefore be interpreted primarily in the context of clinically vulnerable hospitalized HFpEF and should not be directly generalized to ambulatory or lower-risk HFpEF populations. Although patients were not excluded because of recurrent HF hospitalization, the analysis was anchored to the first qualifying hospitalization at our center during the study period. A complete history of HF hospitalizations preceding the index event, particularly admissions to other institutions, was not systematically available; therefore, cumulative prior hospitalization burden could not be evaluated as a prognostic exposure. A greater burden of previous HF hospitalization has been associated with worse outcomes in an HFrEF cohort [22], and this limitation should be considered when interpreting the generalizability of the present findings. Patients with active infectious or inflammatory disease were excluded to reduce the influence of major acute non-HF conditions that could independently affect EASIX components, including creatinine, LDH, and platelet count, as well as mortality risk. This approach was intended to reduce confounding from major acute non-HF conditions when evaluating the prognostic information provided by EASIX in hospitalized HFpEF; however, because infection is a common precipitant of acute heart-failure decompensation, this exclusion narrows the clinical spectrum represented and may limit generalizability to unselected hospitalized HFpEF populations. Second, detailed structural and diastolic echocardiographic indices, including E/e′, left atrial volume index, LV mass index, tricuspid regurgitation velocity/pulmonary artery systolic pressure, and formal diastolic dysfunction grade, as well as body mass index, were not uniformly available across the study period. Consequently, H2FPEF and HFA-PEFF scores could not be systematically and reliably reconstructed. This limitation restricts detailed phenotypic characterization of the cohort, and some degree of HFpEF misclassification cannot be excluded. Although BNP is an established natriuretic peptide biomarker in heart failure, the lack of systematically available NT-proBNP measurements limits direct comparison with studies and prognostic models based on NT-proBNP and may reduce cross-study comparability. Third, the indices were derived from a single set of admission laboratory measurements. As demonstrated by PARAGON-HF and PURSUIT-HFpEF, nutritional-risk measures may change substantially over time [19,20]; serial EASIX trajectories may likewise carry additional prognostic information but were not available in the present study. Although major pharmacological treatment classes recorded during the index hospitalization were available, systematic information on discharge medication regimens, subsequent treatment changes, and longitudinal treatment exposure was not available. Because SGLT2 inhibitors, MRAs, and RAASi/ARNI may influence renal function and clinical outcomes, residual confounding related to treatment exposure cannot be excluded, particularly given that creatinine is a component of EASIX. Treatment patterns may also have evolved during the study period, and residual confounding related to temporal changes in HFpEF management cannot be excluded; however, additional adjustment for calendar year did not materially alter the primary model. Fourth, EASIX is an indirect laboratory composite and cannot establish the presence or severity of endothelial or coronary microvascular dysfunction. Direct vascular assessments, including CAVI and nitric oxide–related biomarkers, were not systematically performed and were therefore unavailable for analysis. Consequently, the relationship between EASIX and directly measured vascular function could not be examined in this cohort. Fifth, only all-cause mortality was available as a reliably ascertainable endpoint. Cause-specific mortality was not systematically adjudicated; therefore, cardiovascular death, including sudden cardiac death, arrhythmic death, and death attributable to worsening heart failure, could not be evaluated separately. Although all-cause mortality avoids potential misclassification of cause of death in a retrospective study, it does not allow determination of whether the prognostic association of EASIX is driven predominantly by heart-failure-related or other cardiovascular mortality rather than by noncardiovascular causes. Finally, all ROC-derived thresholds were identified and evaluated within the same cohort and should therefore be regarded as exploratory rather than clinical decision thresholds. No external validation was performed, and the small number of patients remaining at risk at 5 years resulted in wide confidence intervals around late time-dependent AUC estimates. These findings require confirmation in larger, prospectively characterized HFpEF populations with external validation and serial biomarker assessment.

## 5. Conclusions

In this selected cohort of patients hospitalized with HFpEF, EASIX was nonlinearly associated with long-term all-cause mortality and improved prognostic model fit beyond age, serum sodium, and BNP. PNI was also prognostically informative but provided limited additional information after EASIX was incorporated, whereas CONUT did not improve the model. Given the retrospective, single-center design and modest sample size, these findings should be considered exploratory and hypothesis-generating. Larger prospective studies with external validation are required to determine the reproducibility and potential clinical utility of EASIX in HFpEF.

## Figures and Tables

**Figure 1:**
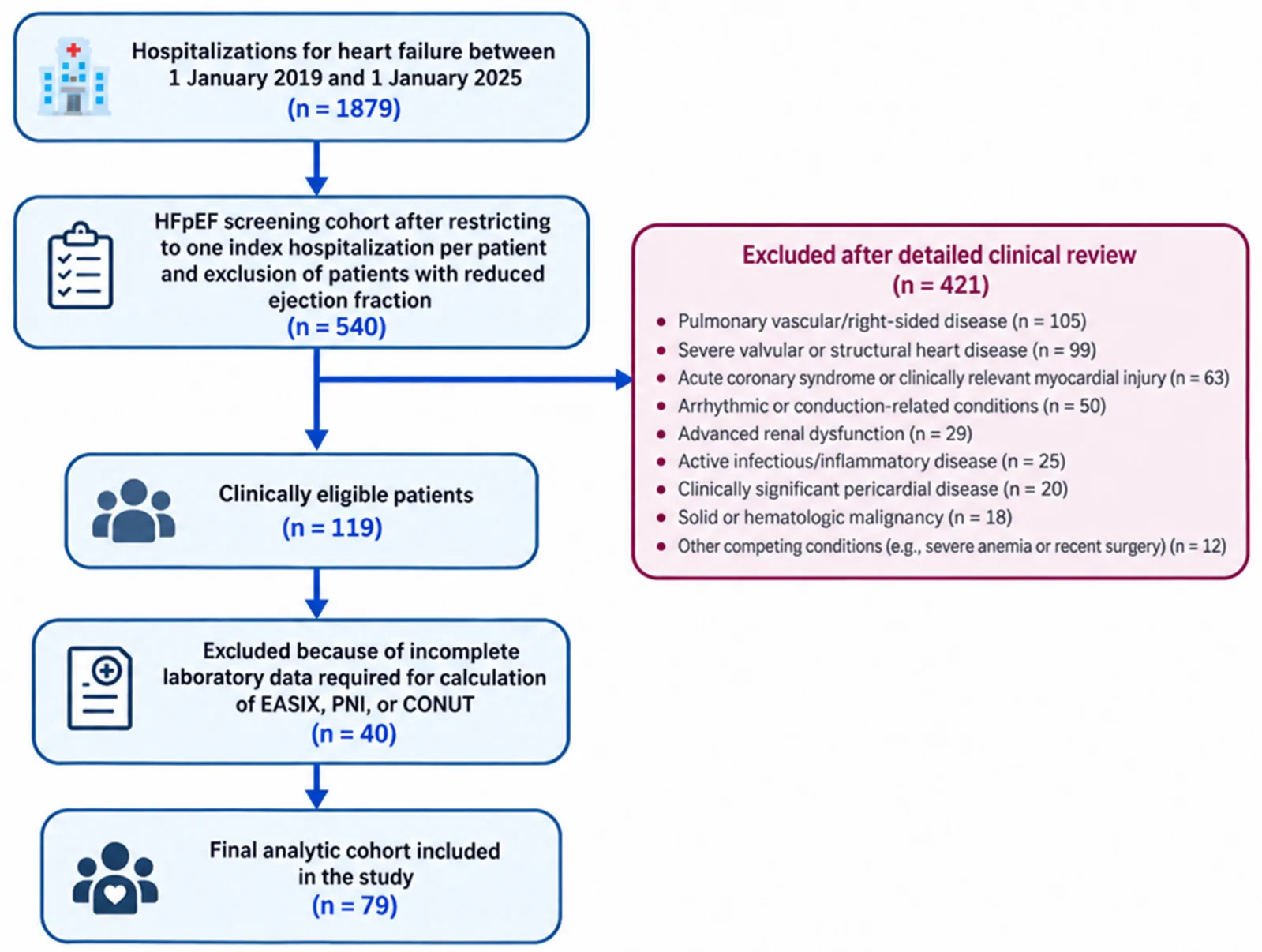
Study flow diagram. Flow diagram illustrating patient selection from 1879 heart failure–related hospitalizations to the final analytic cohort of 79 patients with HFpEF. After restricting the dataset to one index hospitalization per patient and excluding patients with reduced ejection fraction, 540 patients underwent detailed clinical review. Patients with major alternative cardiac or systemic conditions were excluded, leaving 119 clinically eligible patients; 40 additional patients were excluded because of incomplete laboratory data required for calculation of EASIX, PNI, or CONUT.

**Figure 2:**
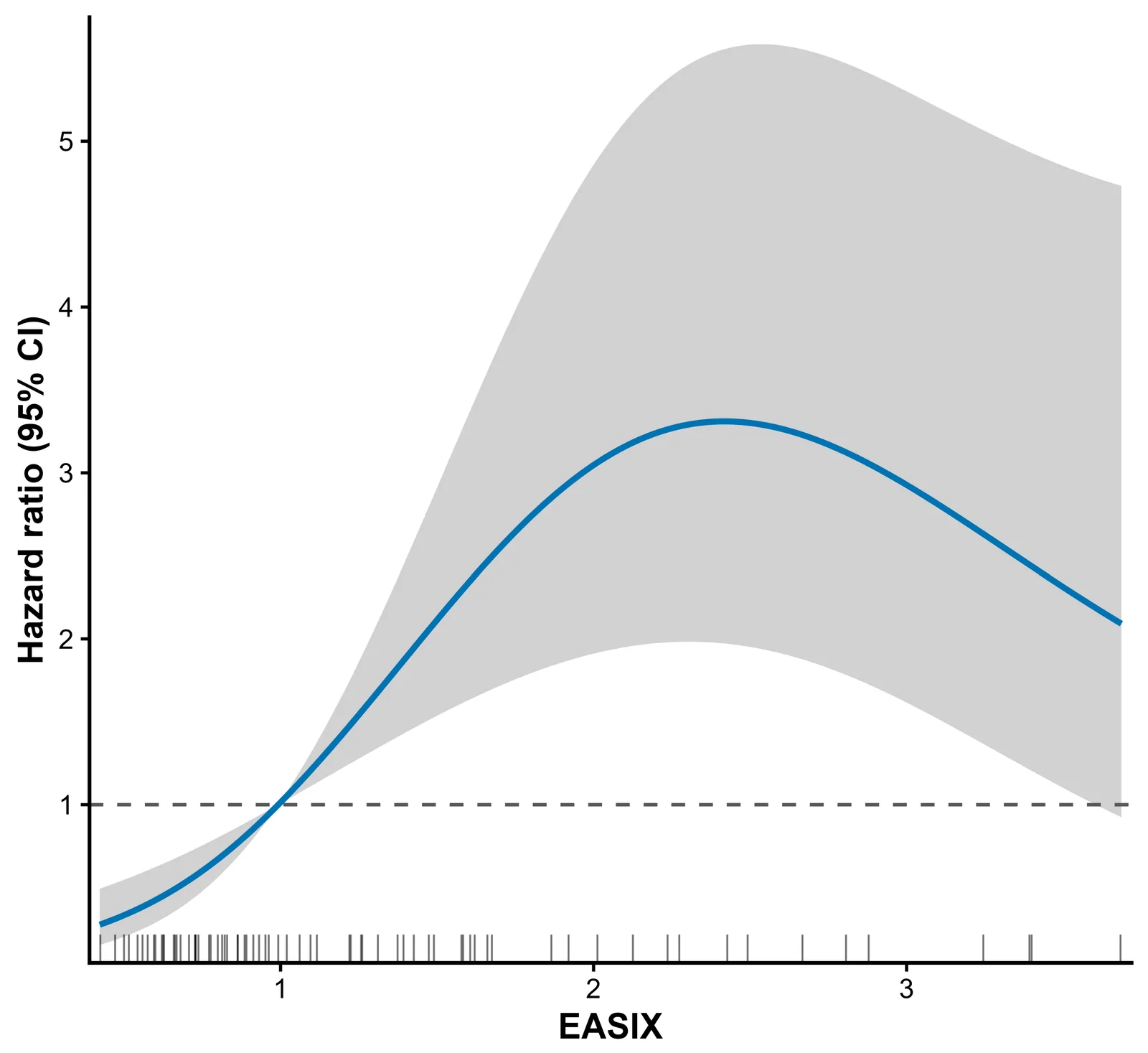
Restricted cubic spline association between EASIX and long-term all-cause mortality. Adjusted hazard ratios for all-cause mortality across the distribution of EASIX were estimated from the primary Cox model incorporating age, serum sodium, BNP, and EASIX modeled using a restricted cubic spline with three knots. The median EASIX value was used as the reference (hazard ratio = 1). The solid line represents the estimated hazard ratio, the dotted line indicates a hazard ratio of 1, the shaded area indicates the 95% confidence interval, and rug marks show the distribution of observed EASIX values. The plot is restricted to the 5th–95th percentile range of EASIX.

**Figure 3:**
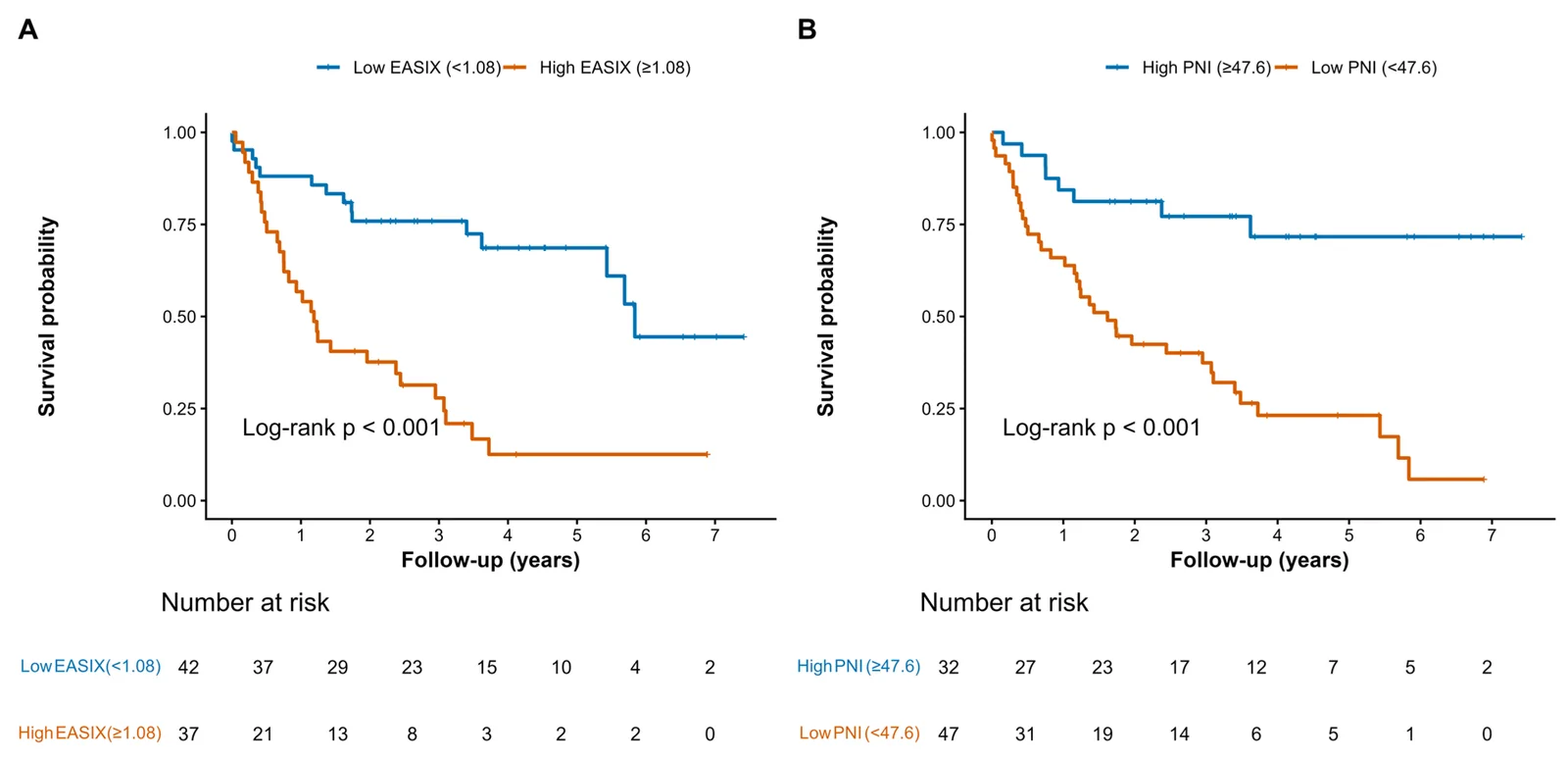
Kaplan–Meier survival curves according to EASIX and PNI. Kaplan–Meier estimates of long-term survival according to ROC-derived EASIX and PNI thresholds. (**A**) Survival according to EASIX < 1.08 versus ≥1.08. (**B**) Survival according to PNI ≥ 47.6 versus <47.6. Survival distributions were compared using the log-rank test, and numbers at risk are shown below each panel. Because the thresholds were derived and evaluated within the same cohort, these analyses were considered exploratory and should not be interpreted as externally validated clinical cut points.

**Figure 4:**
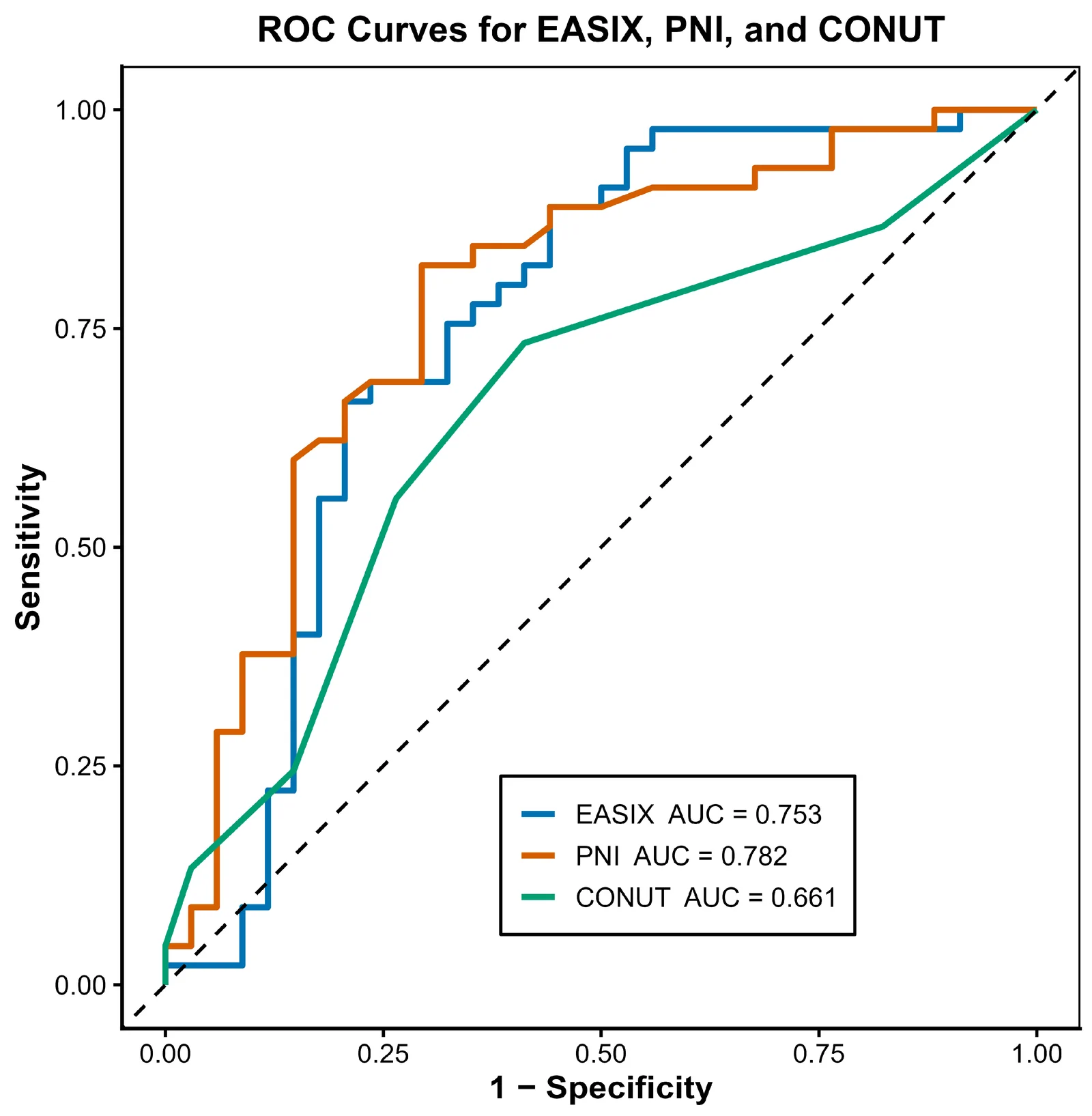
Conventional receiver operating characteristic curves for EASIX, PNI, and CONUT in relation to all-cause mortality. Receiver operating characteristic curves illustrating the conventional discrimination of EASIX, PNI, and CONUT for overall mortality status. The corresponding AUCs were 0.753 for EASIX, 0.782 for PNI, and 0.661 for CONUT. Pairwise differences between correlated AUCs were evaluated using the DeLong method. The dashed diagonal line represents chance discrimination (AUC = 0.5).

**Table 1:** Baseline Clinical and Laboratory Characteristics According to Vital Status.

Characteristic	Overall N = 79	Survivors N = 34	Non-Survivors N = 45	*p* Value
Age, years	72.9 ± 10.4	70.9 ± 8.4	74.5 ± 11.5	0.113
Female sex, n (%)	39 (49%)	19 (56%)	20 (44%)	0.367
Hypertension, n (%)	62 (78%)	29 (85%)	33 (73%)	0.272
Diabetes mellitus, n (%)	39 (49%)	17 (50%)	22 (49%)	>0.999
Dyslipidemia, n (%)	16 (20%)	5 (15%)	11 (24%)	0.399
Coronary artery disease, n (%)	33 (42%)	10 (29%)	23 (51%)	0.067
Current smoking, n (%)	14 (18%)	11 (32%)	3 (6.7%)	0.006
Atrial fibrillation, n (%)	22 (28%)	10 (29%)	12 (27%)	0.805
COPD/asthma, n (%)	22 (28%)	7 (21%)	15 (33%)	0.311
Previous stroke, n (%)	5 (6.3%)	1 (2.9%)	4 (8.9%)	0.384
Chronic kidney disease, n (%)	12 (15%)	2 (5.9%)	10 (22%)	0.060
NYHA class III–IV, n (%)	30 (38%)	11 (32%)	19 (42%)	0.483
LVEF, %	56.8 ± 4.2	57.8 ± 3.6	56.1 ± 4.5	0.067
Pharmacological treatment				
Beta-blocker, n (%)	54 (68.4%)	26 (76.5%)	28 (62.2%)	0.225
RAASi/ARNI, n (%)	43 (54.4%)	24 (70.6%)	19 (42.2%)	0.014
MRA, n (%)	13 (16.5%)	4 (11.8%)	9 (20.0%)	0.376
SGLT2 inhibitor, n (%)	9 (11.4%)	5 (14.7%)	4 (8.9%)	0.488
BNP, pg/mL	270.0 [164.0–579.0]	209.5 [153.0–315.0]	429.0 [196.0–775.0]	0.005
Sodium, mmol/L	138 [135–140]	139 [137–141]	137 [134–139]	0.031
Chloride, mmol/L	104.0 [99.0–107.0]	104.5 [101.0–107.0]	103.0 [97.0–107.0]	0.100
Creatinine, mg/dL	1.0 [0.9–1.5]	0.9 [0.8–1.1]	1.3 [0.9–1.8]	<0.001
eGFR, mL/min/1.73 m2	62.3 ± 24.8	72.7 ± 22.7	54.4 ± 23.6	<0.001
Hemoglobin, g/dL	11.2 ± 1.8	11.8 ± 1.8	10.7 ± 1.6	0.010
Lymphocyte count, ×109/L	1.6 [1.2–2.0]	1.9 [1.4–2.3]	1.5 [1.2–1.8]	0.004
Platelet count, ×109/L	254.7 [196.6–280.0]	262.9 [196.6–280.0]	245.6 [200.1–279.0]	0.683
CRP, mg/L	8.7 [2.9–18.6]	6.3 [2.7–10.3]	10.9 [4.3–23.5]	0.015
Albumin, g/L	38 [34–42]	41 [38–43]	36 [33–39]	<0.001
Total cholesterol, mg/dL	168.6 ± 42.5	166.8 ± 35.7	170.0 ± 47.3	0.732
LDH, U/L	220 [178–283]	192 [170–240]	237 [197–286]	0.005
EASIX	0.99 [0.68–1.68]	0.72 [0.56–0.99]	1.39 [0.89–2.01]	<0.001
Prognostic Nutritional Index	46.1 ± 7.8	50.4 ± 7.4	42.9 ± 6.5	<0.001
CONUT score	2 [1–4]	1 [1–3]	3 [1–4]	0.013

Data are presented as mean ± SD, median [Q1–Q3], or n (%), as appropriate. *p* values were calculated using Welch’s two-sample *t*-test, Wilcoxon rank-sum test, or Fisher’s exact test, as appropriate. Abbreviations: ARNI, angiotensin receptor–neprilysin inhibitor; BNP, B-type natriuretic peptide; CONUT, Controlling Nutritional Status; COPD, chronic obstructive pulmonary disease; CRP, C-reactive protein; EASIX, Endothelial Activation and Stress Index; eGFR, estimated glomerular filtration rate; LDH, lactate dehydrogenase; LVEF, left ventricular ejection fraction; MRA, mineralocorticoid receptor antagonist; NYHA, New York Heart Association; PNI, Prognostic Nutritional Index; Q1, first quartile; Q3, third quartile; RAASi, renin–angiotensin system inhibitor; SD, standard deviation; SGLT2, sodium–glucose cotransporter 2.

**Table 2:** Univariable Cox Regression Analyses for Long-Term All-Cause Mortality.

Characteristic	N	HR	95% CI	*p* Value
Age, per 1 year	79	1.03	1.00–1.06	0.079
Female sex	79	0.66	0.36–1.19	0.165
Hypertension	79	0.86	0.44–1.68	0.669
Diabetes mellitus	79	1.08	0.60–1.95	0.790
Dyslipidemia	79	1.44	0.73–2.86	0.291
Coronary artery disease	79	1.73	0.96–3.11	0.067
Current smoking	79	0.26	0.08–0.84	0.024
Atrial fibrillation	79	0.82	0.42–1.60	0.557
COPD/asthma	79	1.63	0.88–3.04	0.124
Previous stroke	79	1.48	0.53–4.15	0.460
Chronic kidney disease	79	2.04	1.01–4.13	0.048
NYHA class III–IV	79	1.38	0.76–2.50	0.283
LVEF, per 1%	79	0.92	0.86–0.99	0.029
BNP, per 100 pg/mL	79	1.07	1.03–1.12	<0.001
Sodium, per 1 mmol/L	79	0.94	0.89–0.99	0.022
Chloride, per 1 mmol/L	79	0.95	0.91–0.99	0.025
Creatinine, per 1 mg/dL	79	2.18	1.47–3.23	<0.001
eGFR, per 10 mL/min/1.73 m^2^	79	0.79	0.70–0.90	<0.001
Hemoglobin, per 1 g/dL	79	0.79	0.66–0.95	0.011
Lymphocyte count, per 1 × 10^9^/L	79	0.60	0.39–0.92	0.020
Platelet count, per 50 × 10^9^/L	79	0.95	0.81–1.12	0.550
CRP, per 10 mg/L	79	1.07	0.98–1.18	0.138
Albumin, per 5 g/L	79	0.58	0.45–0.75	<0.001
Total cholesterol, per 10 mg/dL	79	1.01	0.94–1.09	0.782
LDH, per 100 U/L	79	1.56	1.20–2.03	0.001
EASIX, per 1-unit increase	79	1.31	1.08–1.60	0.007
PNI, per 5-point increase	79	0.63	0.51–0.78	<0.001
CONUT, per 1-point increase	79	1.21	1.05–1.39	0.009

Hazard ratios were estimated using univariable Cox proportional hazards regression. Continuous variables are expressed per the increment specified in the table. The linear EASIX estimate is presented descriptively; because subsequent restricted cubic spline analysis demonstrated significant nonlinearity, it was not used as the principal estimate in multivariable modeling. Abbreviations: BNP, B-type natriuretic peptide; CI, confidence interval; CONUT, Controlling Nutritional Status; COPD, chronic obstructive pulmonary disease; CRP, C-reactive protein; EASIX, Endothelial Activation and Stress Index; eGFR, estimated glomerular filtration rate; HR, hazard ratio; LDH, lactate dehydrogenase; LVEF, left ventricular ejection fraction; NYHA, New York Heart Association; PNI, Prognostic Nutritional Index.

**Table 3:** Multivariable Prognostic Models, Incremental Model Comparisons, and Internal Validation.

Panel (**A**) Multivariable Cox Regression Models				
**Variable**	**Primary Model HR (95% CI)**	***p* Value**	**Extended Model HR (95% CI)**	***p* Value**
Age, per 1 year	1.03 (0.99–1.06)	0.102	1.03 (0.99–1.06)	0.103
Sodium, per 1 mmol/L	0.93 (0.88–0.98)	0.010	0.94 (0.89–0.99)	0.021
BNP, per 100 pg/mL	1.08 (1.03–1.13)	0.003	1.05 (1.00–1.11)	0.063
EASIX, restricted cubic spline (3 knots)	—	<0.001	—	<0.001
Nonlinear component	—	<0.001	—	<0.001
PNI, per 5-point increase	—	—	0.78 (0.59–1.02)	0.066
Panel (**B**) Incremental Prognostic Value of EASIX, PNI, and CONUT				
**Comparison**	**LR χ^2^**	**df**	***p* Value**	
Clinical → + PNI	8.74	1	0.003	
Clinical → + EASIX	24.36	2	<0.001	
Clinical + EASIX → + PNI	3.54	1	0.060	
Clinical + PNI → + EASIX	19.16	2	<0.001	
Clinical + EASIX → + CONUT	0.03	1	0.859	
Panel (**C**) Model Performance and Internal Validation				
**Model**	**Apparent C-Index**	**Optimism-Corrected C-Index**	**Optimism-Corrected Calibration Slope**	**AIC**
Clinical + EASIX	0.763	0.728	0.729	317.25
Clinical + EASIX + PNI	0.774	0.732	0.667	315.71

The clinical reference model included age, serum sodium, and BNP. The primary model additionally included EASIX modeled using a restricted cubic spline with three knots; the extended model further included PNI. For spline-modeled EASIX, *p* values represent joint tests of the spline terms, and a single HR is therefore not reported. Internal validation was performed using 1000 bootstrap resamples. Abbreviations: AIC, Akaike information criterion; BNP, B-type natriuretic peptide; CI, confidence interval; CONUT, Controlling Nutritional Status; EASIX, Endothelial Activation and Stress Index; HR, hazard ratio; LR, likelihood ratio; PNI, Prognostic Nutritional Index.

**Table 4:** Conventional ROC Analysis and Pairwise Comparison of EASIX, PNI, and CONUT for All-Cause Mortality.

Panel (**A**) Conventional ROC analysis					
**Marker**	**AUC**	**95% CI**	**Optimal Cutoff**	**Sensitivity**	**Specificity**
EASIX	0.753	0.634–0.872	1.078	66.7%	79.4%
PNI	0.782	0.675–0.889	47.625	82.2%	70.6%
CONUT	0.661	0.539–0.782	≥2	73.3%	58.8%
Panel (**B**) Pairwise DeLong comparisons					
**Comparison**	**ΔAUC**	**95% CI for ΔAUC**	**Z**	***p* Value**	
EASIX vs. PNI	−0.029	−0.188 to 0.130	−0.359	0.719	
EASIX vs. CONUT	0.092	−0.073 to 0.257	1.097	0.273	
PNI vs. CONUT	0.121	0.043 to 0.200	3.028	0.002	

Optimal cutoffs were determined using the Youden index. ΔAUC was calculated as the AUC of the first marker minus that of the second marker. Pairwise AUC comparisons were performed using the DeLong method. Abbreviations: AUC, area under the curve; CI, confidence interval; CONUT, Controlling Nutritional Status; EASIX, Endothelial Activation and Stress Index; PNI, Prognostic Nutritional Index; ROC, receiver operating characteristic.

**Table 5:** Time-Dependent Discrimination of EASIX, PNI, and CONUT for All-Cause Mortality.

Panel (**A**) Time-dependent AUCs			
**Marker**	**1-Year AUC (95% CI)**	**3-Year AUC (95% CI)**	**5-Year AUC (95% CI)**
EASIX	0.745 (0.624–0.867)	0.760 (0.635–0.884)	0.803 (0.620–0.986)
PNI	0.613 (0.485–0.742)	0.725 (0.604–0.847)	0.805 (0.676–0.934)
CONUT	0.516 (0.365–0.667)	0.607 (0.474–0.739)	0.641 (0.480–0.802)
Panel (**B**) Pairwise time-dependent AUC comparisons			
**Comparison**	**1-Year Adjusted *p* Value**	**3-Year Adjusted *p* Value**	**5-Year Adjusted *p* Value**
EASIX vs. PNI	0.397	0.954	1.000
EASIX vs. CONUT	0.091	0.192	0.242
PNI vs. CONUT	0.160	0.024	0.002
Panel (**C**) Holm-Adjusted Sensitivity Analysis Across All Comparisons			
**Comparison**	**Time**	**Holm-Adjusted *p* Value**	
EASIX vs. PNI	1 year	0.549	
EASIX vs. PNI	3 years	1.000	
EASIX vs. PNI	5 years	1.000	
EASIX vs. CONUT	1 year	0.267	
EASIX vs. CONUT	3 years	0.438	
EASIX vs. CONUT	5 years	0.442	
PNI vs. CONUT	1 year	0.438	
PNI vs. CONUT	3 years	0.075	
PNI vs. CONUT	5 years	0.008	

Time-dependent AUCs were estimated using inverse probability of censoring weighting. *p* values in Panel (**B**) were adjusted using the Holm method across the three time points within each marker pair. Panel (**C**) presents a more conservative Holm adjustment applied jointly across all nine pairwise time-specific comparisons. Five-year estimates should be considered exploratory because only 12 patients remained at risk at 5 years. Abbreviations: AUC, area under the curve; CI, confidence interval; CONUT, Controlling Nutritional Status; EASIX, Endothelial Activation and Stress Index; PNI, Prognostic Nutritional Index.

## Data Availability

The datasets generated and/or analyzed during the current study are not publicly available due to ethical and privacy restrictions but may be available from the corresponding author on reasonable request, subject to applicable institutional and ethical requirements.

## Supplementary Materials

The following supporting information can be downloaded at: https://www.mdpi.com/article/10.3390/biomedicines14102228/s1, Figure S1: Three-year calibration of the primary and extended prognostic models. Calibration plots compare model-predicted 3-year survival probabilities with observed survival for (A) the clinical + EASIX model and (B) the clinical + EASIX + PNI model. Black circles connected by solid lines represent apparent Kaplan–Meier estimates, with vertical bars indicating 95% confidence intervals; blue crosses represent bootstrap optimism-corrected calibration estimates obtained from 1000 bootstrap resamples. The solid lines are included only as visual guides between the apparent grouped estimates. The dashed diagonal line represents ideal calibration. Rug marks along the upper margin indicate the distribution of individual predicted 3-year survival probabilities.

## Abbreviations

The following abbreviations are used in this manuscript:

AIC	Akaike information criterion
AUC	area under the curve
BNP	B-type natriuretic peptide
C-index	concordance index
CI	confidence interval
CONUT	Controlling Nutritional Status
CRP	C-reactive protein
df	degrees of freedom
EASIX	Endothelial Activation and Stress Index
eGFR	estimated glomerular filtration rate
GNRI	Geriatric Nutritional Risk Index
H2FPEF	Heavy, Hypertensive, Atrial Fibrillation, Pulmonary Hypertension, Elder, and Filling Pressure
HF	heart failure
HFA-PEFF	Heart Failure Association Pre-test Assessment, Echocardiography and Natriuretic Peptide, Functional Testing, and Final Etiology
HFmrEF	heart failure with mildly reduced ejection fraction
HFpEF	heart failure with preserved ejection fraction
HFrEF	heart failure with reduced ejection fraction
HR	hazard ratio
IQR	interquartile range
LDH	lactate dehydrogenase
LVEF	left ventricular ejection fraction
NT-proBNP	N-terminal pro-B-type natriuretic peptide
NYHA	New York Heart Association
PARAGON-HF	Prospective Comparison of ARNI with ARB Global Outcomes in Heart Failure with Preserved Ejection Fraction
PNI	Prognostic Nutritional Index
PROMIS-HFpEF	Prevalence of Microvascular Dysfunction in Heart Failure with Preserved Ejection Fraction
PURSUIT-HFpEF	Prospective Multicenter Observational Study of Patients with Heart Failure with Preserved Ejection Fraction
Q1	first quartile
Q3	third quartile
ROC	receiver operating characteristic
TOPCAT	Treatment of Preserved Cardiac Function Heart Failure with an Aldosterone Antagonist Trial
