# Supplementary material for: EASIX for Long-Term Prognostication in Hospitalized Heart Failure with Preserved Ejection Fraction: Incremental Value Beyond Clinical Risk Markers
Source: Biomedicines. 2026 Oct 1;14(10):2228. doi: 10.3390/biomedicines14102228 (PMC13701619; doi:10.3390/biomedicines14102228)
Supplement: Supplementary file 1 [file biomedicines-14-02228-s001.zip › biomedicines-4541153-supplementary.pdf]

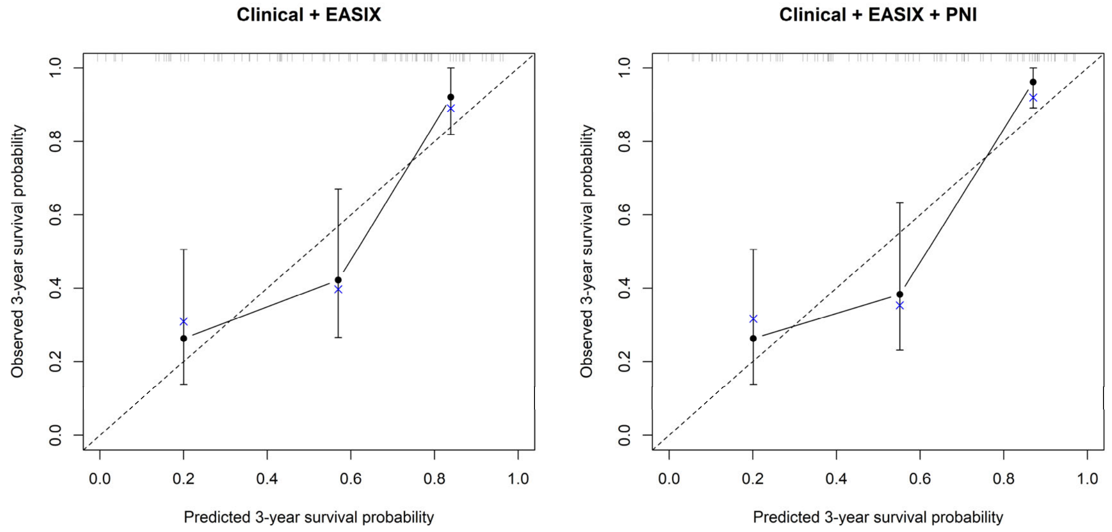


**Supplementary Figure S1. Three-year calibration of the primary and extended prognostic models.** Calibration plots compare model-predicted 3-year survival probabilities with observed survival for (A) the clinical + EASIX model and (B) the clinical + EASIX + PNI model. Black circles connected by solid lines represent apparent Kaplan–Meier estimates, with vertical bars indicating 95% confidence intervals; blue crosses represent bootstrap optimism-corrected calibration estimates obtained from 1,000 bootstrap resamples. The solid lines are included only as visual guides between the apparent grouped estimates. The dashed diagonal line represents ideal calibration. Rug marks along the upper margin indicate the distribution of individual predicted 3-year survival probabilities.
